# Knowledge, attitude, and practice of patients with osteomyelitis: a cross-sectional study using structural equation modeling

**DOI:** 10.3389/fpubh.2026.1838790

**Published:** 2026-07-10

**Authors:** Lin Zhang, Xuan Deng, Jiaze Peng, Jinglin Li, Fuyin Yang, Yang Yu, Xianpeng Huang, Xuxu Yang, Lidan Yang

**Affiliations:** Department of Orthopedics, Affiliated Hospital of Zunyi Medical University, Zunyi, China

**Keywords:** attitude, cross-sectional study, knowledge, osteomyelitis, practice, structural equation modeling

## Abstract

**Introduction:**

To assess the knowledge, attitude, and practice (KAP) levels among patients with osteomyelitis, analyze their associated factors, and explore the pathways between knowledge, attitude, and practice.

**Methods:**

A cross-sectional study was conducted in a large tertiary hospital from April 1st to June 1st, 2025, enrolling 405 patients with osteomyelitis. A self-administered questionnaire collected demographic and disease-related information and assessed KAP. Cronbach’s *α* tested questionnaire reliability, and confirmatory factor analysis examined construct validity. Univariate analysis, multivariate binary logistic regression with a two-tailed *p* < 0.05 as the statistical significance cut-off, and structural equation modeling (SEM) were used to explore factors associated with KAP and their interrelationship mechanisms.

**Results:**

Mean scores for knowledge, attitude, and practice were 28.56 ± 10.28, 29.08 ± 11.36, and 28.58 ± 11.62, respectively; achievement rates were 32.1, 35.6, and 30.9%. The overall Cronbach’s *α* was 0.962, the KMO value was 0.971, and model fit was good (CMIN/DF = 1.32, RMSEA = 0.028, CFI = 0.984). Multivariate analysis showed that age (*p* = 0.013), income (*p* < 0.001), education (*p* < 0.001), history of diabetes mellitus (*p* = 0.009), and number of hospitalizations (*p* = 0.020) were independently associated with knowledge. Knowledge level (*p* < 0.001), age (*p* = 0.034), income (*p* < 0.001), and education (*p* < 0.001) were associated with attitude. Attitude level (*p* < 0.001), knowledge level (*p* < 0.001), income (*p* < 0.001), and education (*p* = 0.019) were associated with practice. SEM revealed that knowledge had a direct theoretical association with attitude (*β* = 0.694, *p* < 0.001) and practice (*β* = 0.303, *p* < 0.001); attitude had a direct theoretical association with practice (*β* = 0.465, *p* < 0.001). The indirect theoretical association of knowledge with practice via attitude was significant (*β* = 0.357, *p* < 0.001), accounting for 54.1% of the total effect.

**Conclusion:**

Patients with osteomyelitis showed insufficient knowledge, attitude, and practice. Older age, lower education, and lower income were associated with poorer outcomes. Attitude mediated the knowledge-practice association. Future interventions should focus on attitude change in vulnerable populations, and prospective studies are warranted.

## 1 Introduction

Osteomyelitis is an infectious disease of bone tissue caused by bacteria, mycobacteria, or fungi. It is characterized by sinus drainage, sequestrum, and dead space. The disease can affect the periosteum, bone, and bone marrow. It is a common refractory infectious disease in orthopedics. Severe cases can lead to disability and reduce quality of life, imposing a significant social and economic burden (1, 2). Osteomyelitis can arise from hematogenous spread, contiguous spread from adjacent soft tissue infections, or direct inoculation due to trauma or surgery (3). Globally, the epidemiological distribution of osteomyelitis shows significant regional variation. The incidence of childhood osteomyelitis in low-income countries can reach 43–200 cases per 100,000 population. This rate is significantly higher than the 1.94–13 cases per 100,000 in high-income countries (4). This disparity reflects the impact of healthcare accessibility, treatment timeliness, and socioeconomic factors on disease prognosis.

Treating osteomyelitis is challenging. It requires long-term, adequate, and sensitive antibiotic therapy, often combined with surgical debridement. Studies indicate that bone and joint infections require 4–8 weeks of antimicrobial therapy, sometimes extending to several months (5). Adherence to the treatment plan is a critical determinant of osteomyelitis prognosis. Successful treatment requires not only selecting sensitive antibiotics but also ensuring patients strictly follow the long-term regimen. A study in South Africa showed that although 77% of patients self-reported high adherence, most antibiotic prescriptions did not conform to national standard treatment guidelines (6). Insufficient patient knowledge about the disease and medication can lead to improper medication use and treatment interruption. This may increase the risk of treatment failure and antimicrobial resistance. Research indicates that poor patient knowledge of prescribed medications is associated with medication errors. Similarly, a lack of knowledge about one’s condition negatively affects the effectiveness of disease management interventions. A qualitative study in Rwanda reported that 85% of patients delayed seeking care. They initially relied on traditional therapies due to financial difficulties, stigma, and misconceptions about the disease (7). Therefore, improving medication adherence in chronic disease patients through patient education and clinical pharmacist consultation has proven effective.

The Knowledge-Attitude-Practice (KAP) model posits that individual health behaviors are jointly influenced by knowledge and attitudes (8–10). In chronic disease management, KAP surveys help researchers understand how patients’ cognitive levels relate to their behavioral choices. This provides a basis for developing targeted health education interventions. This model has been widely applied in managing various chronic diseases such as coronary heart disease, diabetes mellitus (DM), and cancer (11, 12).

Unlike many acute infections, osteomyelitis often requires prolonged antimicrobial treatment lasting weeks to months, repeated surgical procedures, and continuous follow-up. Successful disease management depends not only on medical interventions but also on patients’ understanding of treatment objectives, adherence to medication regimens, wound-care behaviors, and participation in follow-up monitoring. Poor adherence has been associated with treatment failure, recurrent infection, increased healthcare utilization, and antimicrobial resistance. Despite the recognized importance of patient engagement in chronic infection management, little is known about the knowledge, attitudes, and practices of patients with osteomyelitis. Therefore, applying the KAP framework may provide important insights into behavioral determinants that influence disease management and long-term outcomes. While studies have examined medication adherence or knowledge gaps separately in bone infections, the interrelationship among knowledge, attitude, and practice has not been systematically investigated in this population. Specifically, it remains unclear whether patients’ understanding of osteomyelitis directly correlates with their self-management behaviors, or whether this relationship is mediated by their attitudes toward the disease and its treatment. Based on the theoretical KAP framework, this study aimed to: (1) assess KAP levels among patients with osteomyelitis; (2) analyze demographic and clinical factors associated with KAP levels; and (3) test a hypothesized pathway model using SEM to explore whether attitude mediates the theoretical relationship between knowledge and practice, thereby providing a potential theoretical basis for future clinical care and health education efforts.

## 2 Methods

### 2.1 Study design and participants

We conducted this cross-sectional survey in the orthopedic outpatient clinics and wards of a large tertiary hospital from April 1st to June 1st, 2025. The study population comprised patients diagnosed with osteomyelitis. Inclusion criteria were: (1) age ≥18 years; (2) confirmed diagnosis of osteomyelitis based on clinical and imaging findings, with disease duration ≥3 months; (3) conscious, no communication barriers, and voluntary participation. Exclusion criteria were: (1) concurrent malignant tumor, active tuberculosis, or other severe infectious diseases; (2) intellectual disability or mental illness preventing cooperation with the survey.

This study was approved by the hospital’s ethics committee (ID: KLLY-2024-122). All participants provided signed informed consent.

### 2.2 Sample size estimation

We calculated sample size to meet the requirements of both logistic regression and SEM. For logistic regression, we followed an events-per-variable (EPV) of at least 10. For our primary analysis of practice (30.9% prevalence), with up to 10 predictor variables, we needed a minimum of 10*10/0.309 ≈ 324 cases. For SEM, a common rule of thumb is 10–20 participants per measured variable (30 items), requiring 300–600 cases. To account for invalid responses, we enrolled 430 patients, yielding 405 valid cases. This number exceeds the minimum requirements for both analytical approaches.

### 2.3 Survey instrument

The questionnaire were rigorously derived from clinical guidelines for osteomyelitis diagnosis and treatment and relevant literature (13–16). The draft was reviewed and revised by a panel of experts including two orthopedic surgeons, one infectious disease physician, and two senior nurses to ensure content validity. The questionnaire was then refined through a pilot survey on 30 patients to assess clarity and comprehensibility. The final questionnaire comprised four sections. A score of ≥70% of the maximum possible score (i.e., ≥35 out of 50) was defined as adequate knowledge, a positive attitude, or proactive practice. This 70% threshold is a widely used criterion in health education and KAP research to indicate a passing or satisfactory level of mastery or agreement (17–19).

#### 2.3.1 Demographic and clinical characteristics

We collected data on sex, age, residence, monthly income, education level, smoking history, history of DM, history of underlying diseases or immunosuppression, surgery-related osteomyelitis, and number of osteomyelitis-related hospitalizations.

#### 2.3.2 Knowledge dimension

This dimension consisted of 10 items covering etiology, risk factors, diagnostic indicators, and treatment duration. We used a 5-point Likert scale (1 = very unfamiliar, 5 = very familiar). Total scores ranged from 10 to 50. A score ≥70% (≥35) defined adequate knowledge.

#### 2.3.3 Attitude dimension

This dimension consisted of 10 items assessing patients’ perception of disease severity, confidence in treatment, psychological reactions, and willingness to comply. We used a 5-point Likert scale (1 = strongly disagree, 5 = strongly agree). Total scores ranged from 10 to 50. A score ≥70% (≥35) defined a positive attitude.

#### 2.3.4 Practice dimension

This dimension consisted of 10 items assessing medication adherence, follow-up behavior, wound observation, rehabilitation exercises, and nutritional support. We used a 5-point Likert scale (1 = never, 5 = always). Total scores ranged from 10 to 50. A score ≥70% (≥35) defined proactive practice.

### 2.4 Quality control

We collected questionnaires using a combination of online and offline methods. For outpatient and inpatient participants, trained medical staff provided on-site guidance. For discharged patients followed up, we collected data via telephone or WeChat platform. We embedded quality control questions, including several simple arithmetic problems, in the questionnaire. According to published recommendations on survey quality control (20, 21), completion times significantly below the normal range may indicate random responding. Based on the pilot test of 30 patients, the mean completion time was 162 ± 41 s, and the fastest valid completion took approximately 120 s. Therefore, we set a cutoff of <90 s. We excluded questionnaires completed in <90 s or those with incorrect answers to the quality control questions. All questionnaires were checked for completeness before data entry. Questionnaires containing substantial missing information were excluded; therefore, no missing data were included in the final analyses.

### 2.5 Statistical analysis

SPSS 22.0 and AMOS 24.0 were used for data analysis.

#### 2.5.1 Reliability and validity testing

We assessed internal consistency using Cronbach’s *α* coefficient (α > 0.7 indicates good reliability). We used the Kaiser-Meyer-Olkin (KMO) test and Bartlett’s test of sphericity to evaluate suitability for factor analysis (KMO > 0.8 indicates good suitability). We used confirmatory factor analysis (CFA) to test construct validity, with model fit indices including CMIN/DF, RMSEA, IFI, TLI, and CFI.

#### 2.5.2 Univariate analysis

We used independent t-tests or analysis of variance (ANOVA) to compare KAP scores among patients with different characteristics.

#### 2.5.3 Multivariate analysis

We included variables with *p* < 0.10 in the univariate analyses as independent variables in the binary logistic regression models to reduce the risk of excluding potentially important variables. For logistic regression analyses, KAP scores were dichotomized based on the predefined 70% threshold. Participants scoring ≥70% of the maximum attainable score were classified as having adequate knowledge, positive attitude, or proactive practice, whereas those scoring <70% were classified as non-achievers. These dichotomized variables were used as dependent variables in subsequent logistic regression analyses. We calculated odds ratios (OR) and 95% confidence intervals (CI).

#### 2.5.4 Structural equation Modeling

While logistic regression identifies independent associations between factors and KAP levels, we used SEM to test a theoretically hypothesized pathway model based on the KAP framework. In this model, we specified knowledge as the exogenous latent variable, attitude as the mediating variable, and practice as the endogenous latent variable. We chose SEM because it allows simultaneous estimation of direct and indirect pathways, quantification of the mediating effect of attitude, and accounting for measurement error in the latent variables. We used the maximum likelihood method to estimate path coefficients and the bootstrap method to test the significance of the mediating effect. We set the significance level at *α* = 0.05.

While logistic regression identified independent factors associated with KAP achievement, SEM allowed examination of the theoretical relationships among latent KAP constructs. The added value of SEM lies in its ability to simultaneously evaluate direct and indirect associations among knowledge, attitudes, and practices within a unified conceptual framework, thereby providing additional insights beyond conventional regression analyses.

## 3 Results

### 3.1 Sample demographic and clinical characteristics

We included 405 patients with osteomyelitis. There were 246 males (60.74%) and 159 females (39.26%). Age distribution was predominantly 30–50 years (34.32%) and 51–70 years (31.85%), with <30 years accounting for 22.72% and >70 years for 11.11%. Urban residents numbered 232 (57.28%), and rural residents 173 (42.72%). Monthly income was mainly 2000–5,000 RMB (31.60%) and 5,000–10,000 RMB (26.17%), with <2000 RMB accounting for 20.25%, 10,000–20,000 RMB for 15.80%, and >20,000 RMB for 6.17%. Regarding education level, most patients had junior high school or below (65.19%), followed by high school/vocational school (23.70%), and bachelor’s degree or above (11.11%). Smokers comprised 33.09%, those with a history of DM comprised 34.57%, and those with a history of underlying diseases or immunosuppression comprised 42.96%. Surgery-related osteomyelitis accounted for 60.99%, and non-surgery-related for 39.01%. The number of osteomyelitis-related hospitalizations was predominantly 1 (37.53%), with 0 times accounting for 23.21%, 2 times for 18.77%, and ≥3 times for 20.49% (Table 1).

**Table 1:** General demographic and disease-related characteristics of study participants.

Variable	Category	Frequency	Percentage (%)
Sex	Male	246	60.741
	Female	159	39.259
Age (years)	<30	92	22.716
	30–50	139	34.321
	51–70	129	31.852
	>70	45	11.111
Residence	Rural	173	42.716
	Urban	232	57.284
Monthly income (RMB)	<2000	82	20.247
	2000–5,000	128	31.605
	5,000–10,000	106	26.173
	10,000–20,000	64	15.802
	>20,000	25	6.173
Education level	Junior high or below	264	65.185
	High school/Vocational	96	23.704
	Bachelor’s or above	45	11.111
Smoking	Yes	134	33.086
	No	271	66.914
History of diabetes	Yes	140	34.568
	No	265	65.432
History of underlying disease/immunosuppression	Yes	174	42.963
	No	231	57.037
Surgery-related osteomyelitis	Yes	247	60.988
	No	158	39.012
Number of osteomyelitis-related hospitalizations	0	94	23.21
	1	152	37.531
	2	76	18.765
	>3	83	20.494
Total		405	100

### 3.2 Reliability and validity testing

The overall Cronbach’s *α* coefficient for the questionnaire was 0.962. The Cronbach’s α coefficients for the knowledge, attitude, and practice dimensions were 0.922, 0.941, and 0.943, respectively, indicating excellent reliability. The KMO value was 0.971, and Bartlett’s test of sphericity yielded an approximate chi-square value of 8435.771 (df = 435, *p* < 0.001). These results indicated that the data were highly suitable for factor analysis. The rotated factor loading matrix showed that each item’s loading on its corresponding dimension was above 0.6 (knowledge dimension: 0.668–0.725, attitude dimension: 0.703–0.739, practice dimension: 0.701–0.763). All communalities were greater than 0.5, indicating good explanatory power of each dimension. Harman’s single-factor test extracted three factors with eigenvalues greater than 1, explaining 63.627% of the total variance. No single factor explained most of the variance, suggesting no significant common method bias.

Confirmatory factor analysis (CFA) showed good model fit: CMIN/DF = 1.32, RMSEA = 0.028, IFI = 0.984, TLI = 0.983, CFI = 0.984. Factor loadings for each item ranged from 0.697 to 0.809 (all *p* < 0.001). Composite reliability (CR) values were 0.923, 0.941, and 0.943, respectively. Average variance extracted (AVE) values were 0.544, 0.615, and 0.625, respectively, all meeting recommended standards. The square roots of AVE for each dimension (knowledge 0.738, attitude 0.784, practice 0.791) were greater than the inter-dimensional correlations (knowledge-attitude *r* = 0.645, knowledge-practice *r* = 0.583, attitude-practice *r* = 0.636). These results indicated good convergent and discriminant validity.

### 3.3 Knowledge, attitude, practice scores and univariate analysis

The mean knowledge score was 28.56 ± 10.28, with an achievement rate of 32.1%; the mean attitude score was 29.08 ± 11.36, with an achievement rate of 35.6%; the mean practice score was 28.58 ± 11.62, with an achievement rate of 30.9%. Univariate analysis results (Table 2) showed significant differences in KAP scores among patients with different characteristics.

**Table 2:** Univariate comparison of knowledge, attitude, and practice scores among different population subgroups.

Variable	Category	Knowledge		Attitude		Practice	
		Mean	SD	Mean	SD	Mean	SD
Sex	Male	28.886	10.182	29.598	11.237	29.22	11.547
	Female	27.472	10.072	28.151	11.036	27.346	11.519
	*t*	1.371		1.274		1.596	
	*p*	0.171		0.203		0.111	
Age (years)	<30	30.304	11.45	31.543	12.078	31.978	12.428
	30–50	28.669	9.373	29.95	10.457	29.058	11.558
	51–70	28.147	9.559	28.465	10.65	27.364	10.356
	>70	23.778	10.18	22.667	10.592	22.778	10.615
	*F*	4.349		7.162		7.303	
	*p*	<0.05		<0.001		<0.001	
Residence	Rural	26.728	10.939	26.965	11.452	26.509	11.785
	Urban	29.526	9.366	30.569	10.718	29.957	11.186
	*t*	−2.705		−3.25		−2.999	
	*p*	<0.05		<0.05		<0.05	
Income (RMB)	<2000	23.939	8.509	23.744	9.91	24.329	10.291
	2000–5,000	24.773	9.202	25.828	10.602	25.789	11.622
	5,000–10,000	30.491	8.904	32.17	10.193	31.472	10.338
	10,000–20,000	34.016	10.227	33.844	10.328	32.203	12.138
	>20,000	37.24	9.875	37.12	10.713	33.72	10.937
	*F*	22.875		18.228		9.9	
	*p*	<0.001		<0.001		<0.001	
Education level	Junior high or below	25.28	9.326	25.564	10.237	24.852	10.641
	High school/Vocational	30.927	7.493	32.906	8.862	31.625	9.14
	Bachelor’s or above	40.689	8.839	41.089	9.772	43.089	7.119
	*F*	63.366		57.172		70.589	
	*p*	<0.001		<0.001		<0.001	
Smoking	Yes	26.634	9.96	27.112	11.02	26.06	11.364
	No	29.17	10.157	29.978	11.138	29.683	11.486
	*t*	−2.379		−2.445		−2.997	
	*p*	<0.018		<0.015		<0.05	
History of diabetes	Yes	30.2	11.101	29.95	12.291	29.064	12.006
	No	27.343	9.484	28.543	10.517	28.177	11.326
	*t*	2.587		1.15		0.734	
	*p*	0.01		0.251		0.463	
History of underlying disease/immunosuppression	Yes	29.184	10.578	29.73	11.529	28.718	11.864
	No	27.688	9.789	28.502	10.882	28.307	11.345
	*t*	1.47		1.096		0.354	
	*p*	0.142		0.274		0.724	
Surgery-related osteomyelitis	Yes	29.158	10.435	29.814	11.313	29.478	11.682
	No	27.038	9.578	27.804	10.858	26.93	11.223
	*t*	2.058		1.771		2.173	
	*p*	0.04		0.077		<0.1	
Number of osteomyelitis-related hospitalizations	0	25.064	10.102	25.404	11.407	25.745	12.036
	1	28.066	9.733	28.809	11.389	27.882	11.231
	2	29.303	10.101	30.263	10.158	29.421	10.042
	>3	31.627	9.992	32.41	10.27	31.831	12.163
	*F*	6.702		6.414		4.499	
	*p*	<0.001		<0.001		<0.05	

For knowledge scores, significantly higher scores were observed in patients who were younger, resided in urban areas, had higher income, higher education level, were non-smokers, had no history of DM, and had more hospitalizations (*p* < 0.05). Specifically, the knowledge score in the <30 years group (30.30 ± 11.45) was significantly higher than in the >70 years group (23.78 ± 10.18); urban residents scored higher (29.53 ± 9.37) than rural residents (26.73 ± 10.94); the >20,000 RMB income group scored highest (37.24 ± 9.88) compared to the <2000 RMB group (23.94 ± 8.51); the bachelor’s degree or above group scored highest (40.69 ± 8.84) compared to the junior high or below group (25.28 ± 9.33); non-smokers scored higher (29.17 ± 10.16) than smokers (26.63 ± 9.96); patients without DM had lower scores (27.34 ± 9.44) than those with DM (30.20 ± 11.10), a statistically significant difference; patients with ≥3 hospitalizations scored higher (31.63 ± 9.99) than those with 0 hospitalizations (25.06 ± 10.10).

For attitude scores, the effects of age, residence, income, education level, smoking, and number of hospitalizations were statistically significant (*p* < 0.05). The 30–50 years group scored higher (29.95 ± 10.46) than the >70 years group (22.67 ± 10.59); urban residents scored higher (30.57 ± 10.72) than rural residents (26.97 ± 11.45); the >20,000 RMB income group scored highest (37.12 ± 10.71) compared to the <2000 RMB group (23.74 ± 9.91); the bachelor’s degree or above group scored highest (41.09 ± 9.77) compared to the junior high or below group (25.56 ± 10.24); non-smokers scored higher (29.98 ± 11.14) than smokers (27.11 ± 11.03); patients with ≥3 hospitalizations scored higher (32.41 ± 10.27) than those with 0 hospitalizations (25.40 ± 11.41).

For practice scores, the effects of age, residence, income, education level, smoking, surgery-relatedness, and number of hospitalizations were significant (*p* < 0.05). The <30 years group scored higher (31.98 ± 12.43) than the >70 years group (22.78 ± 10.62); urban residents scored higher (29.96 ± 11.19) than rural residents (26.51 ± 11.79); the >20,000 RMB income group scored highest (33.72 ± 10.94) compared to the <2000 RMB group (24.33 ± 10.29); the bachelor’s degree or above group scored highest (43.09 ± 7.12) compared to the junior high or below group (24.85 ± 10.64); non-smokers scored higher (29.68 ± 11.49) than smokers (26.06 ± 11.36); patients with surgery-related osteomyelitis scored higher (29.48 ± 11.68) than those with non-surgery-related osteomyelitis (26.93 ± 11.22); patients with ≥3 hospitalizations scored higher (31.83 ± 12.16) than those with 0 hospitalizations (25.75 ± 12.04).

### 3.4 Multivariate logistic regression analysis

Using knowledge, attitude, and practice as dependent variables, variables with *p* < 0.10 in univariate analyses were included in multivariate logistic regression models.

#### 3.4.1 Factors influencing knowledge

Multivariate analysis results (Table 3) showed that age was significantly negatively associated with knowledge (*β* = −0.444, OR = 0.641, 95%CI: 0.453 ~ 0.909, *p* = 0.013), indicating that older patients were less likely to have adequate knowledge. Income level (*β* = 0.569, OR = 1.767, 95%CI: 1.384 ~ 2.254, *p* < 0.001) and education level (*β* = 1.012, OR = 2.751, 95%CI: 1.874 ~ 4.040, *p* < 0.001) were significantly positively associated with knowledge; higher income and education levels were associated with a greater probability of adequate knowledge. Patients with a history of DM had a significantly lower likelihood of knowledge compared to those without (*β* = −0.907, OR = 0.404, 95%CI: 0.205 ~ 0.795, *p* = 0.009). The number of osteomyelitis-related hospitalizations was positively associated with knowledge (*β* = 0.312, OR = 1.366, 95%CI: 1.051 ~ 1.775, *p* = 0.020); more hospitalizations were associated with a higher likelihood of adequate knowledge. Residence, smoking, and surgery-related osteomyelitis were not statistically significant in the model (*p* > 0.05), suggesting that the independent effects of these factors on knowledge level were limited after controlling for other variables.

**Table 3:** Multivariate logistic regression analysis of knowledge level.

Variable	*p* value	OR value	OR value 95% CI
Age	0.013	0.641	0.453 ~ 0.909
Residence	0.541	0.845	0.493 ~ 1.449
Income	<0.001	1.767	1.384 ~ 2.254
Education level	<0.001	2.751	1.874 ~ 4.040
Smoking	0.303	1.358	0.759 ~ 2.430
History of diabetes	0.009	0.404	0.205 ~ 0.795
Surgery-related osteomyelitis	0.450	0.806	0.460 ~ 1.412
Number of osteomyelitis-related hospitalizations	0.020	1.366	1.051 ~ 1.775

#### 3.4.2 Factors influencing attitude

After controlling for demographic variables, knowledge level was significantly positively associated with attitude (*β* = 1.276, OR = 3.582, 95%CI: 2.032 ~ 6.314, *p* < 0.001), indicating that individuals with higher knowledge levels were more likely to achieve a positive attitude, suggesting a key role of cognitive foundation in attitude formation. Age was significantly negatively associated with attitude (*β* = −0.303, OR = 0.739, 95%CI: 0.558 ~ 0.977, *p* = 0.034); older patients were less likely to achieve a positive attitude. Income level (*β* = 0.419, OR = 1.521, 95%CI: 1.207 ~ 1.916, *p* < 0.001) and education level (*β* = 0.809, OR = 2.246, 95%CI: 1.524 ~ 3.309, *p* < 0.001) were significantly positively associated with attitude; higher income and education levels were associated with a greater probability of a positive attitude. The number of osteomyelitis-related hospitalizations showed a marginally significant positive association (*β* = 0.237, OR = 1.268, 95%CI: 0.990 ~ 1.623, *p* = 0.060), suggesting that increased hospitalizations might contribute to attitude formation, though it did not reach statistical significance. Residence and smoking were not statistically significant in the model (*p* > 0.05) (Table 4).

**Table 4:** Multivariate logistic regression analysis of attitude level.

Variable	*p* value	OR value	OR value 95% CI
Age	0.034	0.739	0.558 ~ 0.977
Residence	0.118	1.514	0.900 ~ 2.549
Income	<0.001	1.521	1.207 ~ 1.916
Education level	<0.001	2.246	1.524 ~ 3.309
Smoking	0.209	1.42	0.822 ~ 2.455
Number of osteomyelitis-related hospitalizations	0.060	1.268	0.990 ~ 1.623
Knowledge	<0.001	3.582	2.032 ~ 6.314

#### 3.4.3 Factors influencing practice

Multivariate analysis results (Table 5) showed that attitude level was significantly positively associated with practice (*β* = 1.558, OR = 4.749, 95%CI: 2.718 ~ 8.299, *p* < 0.001), representing the strongest correlate of practice, indicating that a more positive attitude was associated with a higher likelihood of achieving proactive practice. Knowledge level was also significantly positively associated with practice (*β* = 1.173, OR = 3.231, 95%CI: 1.771 ~ 5.897, *p* < 0.001), indicating that knowledge independently contributed to promoting practice behaviors even after controlling for attitude. Income level (*β* = 0.455, OR = 1.576, 95%CI: 1.230 ~ 2.019, *p* < 0.001) and education level (*β* = 0.503, OR = 1.654, 95%CI: 1.086 ~ 2.517, *p* = 0.019) were significantly positively associated with practice; higher income and education levels were associated with a greater probability of proactive practice. Age, residence, smoking, surgery-related osteomyelitis, and number of hospitalizations were not significant in the model (*p* > 0.05), suggesting that the independent effects of these variables were relatively limited after controlling for knowledge, attitude, and sociodemographic factors.

**Table 5:** Multivariate logistic regression analysis of practice level.

Variable	*p* value	OR value	OR value 95% CI
Age	0.116	0.787	0.584 ~ 1.061
Residence	0.199	1.436	0.827 ~ 2.493
Income	<0.001	1.576	1.230 ~ 2.019
Education level	0.019	1.654	1.086 ~ 2.517
Smoking	0.328	1.329	0.751 ~ 2.353
Surgery-related osteomyelitis	0.681	0.89	0.511 ~ 1.550
Number of osteomyelitis-related hospitalizations	0.225	1.177	0.905 ~ 1.532
Knowledge	<0.001	3.231	1.771 ~ 5.897
Attitude	<0.001	4.749	2.718 ~ 8.299

### 3.5 Structural equation modeling (SEM)

**Figure 1:**
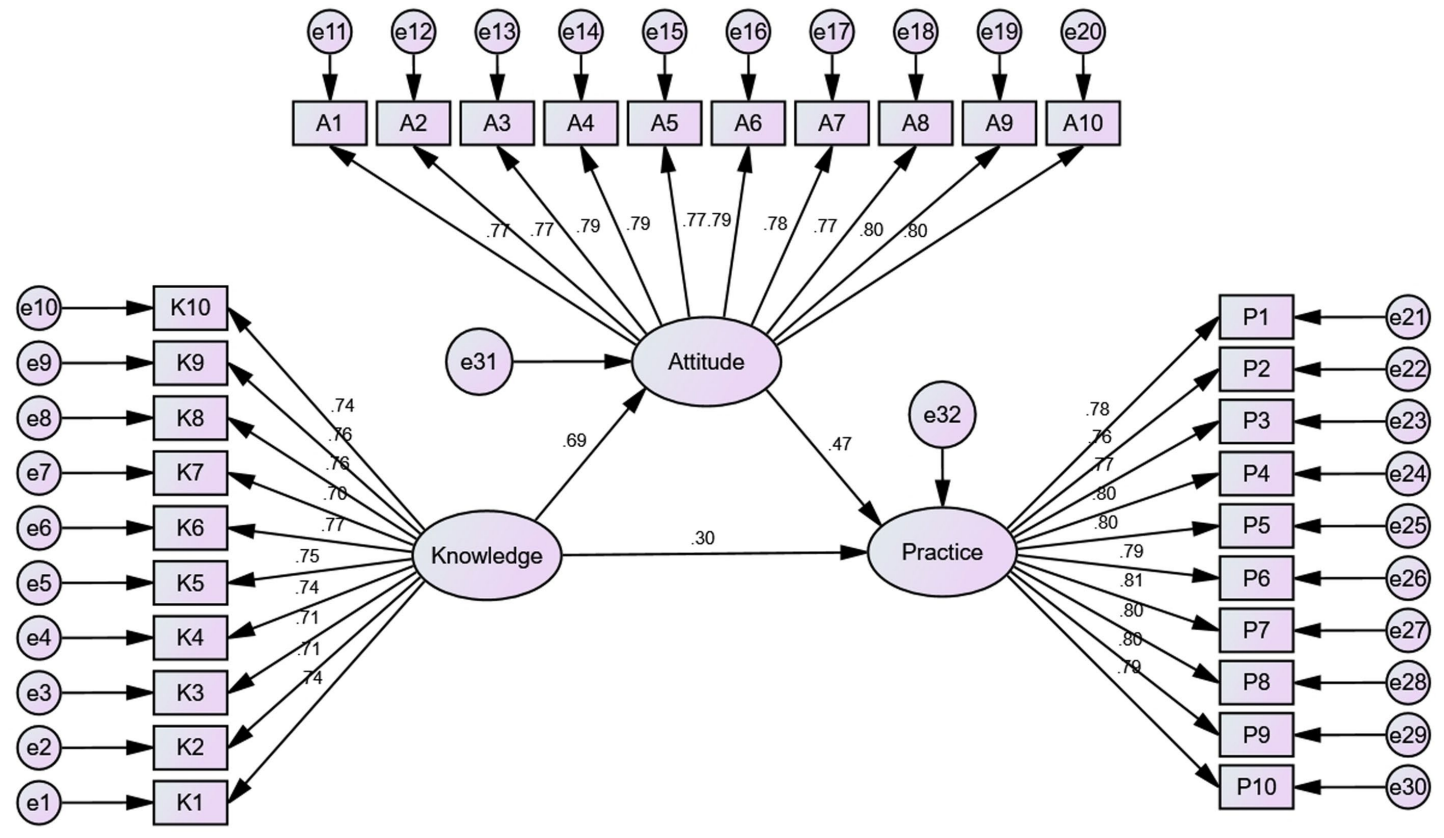
Structural equation model diagram. Structural equation model illustrating the relationships among KAP in patients with osteomyelitis. Standardized path coefficients are presented along the arrows. The model was developed based on the KAP theoretical framework, hypothesizing that knowledge is associated with attitudes and practices, while attitudes may further mediate the association between knowledge and practice.

We constructed a structural equation model with knowledge as the exogenous latent variable, attitude as the mediating variable, and practice as the endogenous latent variable (Figure 1). All model fit indices met the criteria: CMIN/DF = 1.32, RMSEA = 0.028, IFI = 0.984, TLI = 0.983, CFI = 0.984. These values indicated a good fit between the model and the data (Table 6).

**Table 6:** Model fit indices.

Index	Recommended criteria	Actual value	Result
CMIN/DF	1–3 Excellent	1.32	Excellent
RMSEA	<0.05 Excellent, < 0.08 Acceptable	0.028	Excellent
IFI	>0.9 Excellent, > 0.8 Acceptable	0.984	Excellent
TLI	>0.9 Excellent, > 0.8 Acceptable	0.983	Excellent
CFI	>0.9 Excellent, > 0.8 Acceptable	0.984	Excellent

Path analysis results (Table 7) showed that knowledge had a significant positive theoretical association with attitude (*β* = 0.694, *p* < 0.001). Attitude had a significant positive theoretical association with practice (*β* = 0.465, *p* < 0.001). Knowledge had a significant direct theoretical association with practice (*β* = 0.303, *p* < 0.001). This indicates that knowledge was associated with practice both directly and indirectly through attitude.

**Table 7:** Summary of model regression coefficients.

Path	S.E.	C.R.	*p*	Direct effect
Attitude ← Knowledge	0.062	11.826	<0.01	0.694
Practice ← Attitude	0.066	7.36	<0.01	0.465
Practice ← Knowledge	0.067	4.981	<0.01	0.303

The mediation effect test using the bootstrap method showed that the indirect theoretical association of knowledge with practice through attitude was 0.357 (95%CI: 0.233–0.501, *p* < 0.001). Thus, attitude played a partial mediating role between knowledge and practice. The mediation effect accounted for 54.1% of the total effect (indirect effect / total effect = 0.357 / (0.357 + 0.303)) (Table 8).

**Table 8:** Mediation effect test results (bootstrap).

Path	Indirect effect	Lower	Upper	*p*
Knowledge→Attitude→Practice	0.357	0.233	0.501	<0.001

## 4 Discussion

This study systematically assessed KAP levels among patients with osteomyelitis and revealed the structural relationships among these three dimensions using SEM. The results showed that achievement rates for all three dimensions were below 40%. This finding aligns with the clinical reality of persistent poor adherence in chronic bone infections (22–24). The treatment process for osteomyelitis is complex and lengthy. It often requires patients to undergo prolonged antimicrobial therapy and multiple follow-up visits. However, in clinical practice, some patients simplistically understand treatment as “problem solved once surgery is done.” They lack sufficient awareness of the need to complete the full antimicrobial course, the risk of recurrence, and the importance of follow-up. Antimicrobial management studies indicate that patients’ understanding of antibiotic duration and resistance risk is associated with medication adherence (25). In the field of chronic diseases, inadequate health knowledge is considered a significant predictor of treatment failure and hospital readmission (26). Therefore, the low knowledge rate in this study carries an important warning sign. It suggests a significant gap in health education for osteomyelitis patients. This knowledge deficit is a prevalent public health issue, potentially related to multiple factors including the high complexity of the disease, the long treatment cycles, and the accessibility of medical resources.

Multivariate analysis revealed several noteworthy associated factors. Age was a common factor across all three KAP dimensions. Older patients had lower levels of knowledge, attitude, and practice. This phenomenon is consistently observed in various chronic disease KAP studies. For instance, in older patients with type 2 DM, health knowledge, attitudes, and self-management behaviors were significantly lower compared to younger and middle-aged patients. This may be due to decreased information acquisition ability, interference from multiple comorbidities, and ingrained health belief patterns (27). The negative association between diabetes and knowledge achievement may reflect the competing self-management demands faced by patients with multiple chronic conditions. Patients with diabetes are often required to manage complex medication regimens, dietary restrictions, and glycemic monitoring, which may reduce attention to osteomyelitis-specific health information. The associations of education level and income with KAP reflect the central role of health literacy and socioeconomic status in disease management. Consistent evidence shows that higher education and income levels are associated with better knowledge mastery, more positive attitudes, and better practice behaviors (21, 28, 29). Patients with higher education levels typically have stronger abilities to acquire and understand information, enabling more active participation in medical decision-making. Patients with higher income may have better access to healthcare resources and greater capacity to bear treatment burdens. This finding suggests that clinical work should pay particular attention to patients with low education and low income. Clinicians should provide them with easy-to-understand educational materials and resources for financial assistance. The number of hospitalizations was positively correlated with knowledge level. Patients with repeated hospitalizations scored higher on knowledge. This phenomenon may reflect an “experiential learning effect” — during multiple medical encounters, patients acquire more disease-related information from healthcare staff (30, 31). However, this also implies that health education for first-time inpatients or outpatients might be insufficient. This highlights the need to strengthen knowledge transfer during the initial diagnosis and treatment phase. Therefore, enhancing systematic education during the first treatment encounter is of great significance in osteomyelitis management.

The SEM provided the most critical findings of this study. The model revealed three pathways associated with practice: (1) a direct theoretical association of knowledge with practice (*β* = 0.303); (2) a direct theoretical association of attitude with practice (*β* = 0.465); and (3) an indirect theoretical association of knowledge with practice through attitude (*β* = 0.357). This pathway structure supports the KAP theoretical framework and helps elucidate how health behaviors may form in osteomyelitis patients. First, knowledge is theoretically considered a prerequisite for practice. The direct association suggests that patients’ understanding of the disease may translate into health behaviors. For example, patients who understand the need for a full course of antibiotics may be more likely to complete treatment. Second, attitude is a crucial mediator in the knowledge-practice relationship. In this study, the mediating effect of attitude accounted for 54.1% of the total effect. This indicates that nearly half of the knowledge-practice association is explained by attitude. Furthermore, The odds ratio for attitude on practice (OR = 4.749, 95%CI: 2.718–8.299) represents a strong association with clear clinical implications—patients with a positive attitude were nearly five times more likely to report proactive practice behaviors compared to those with a negative attitude. This magnitude of association suggests that interventions aimed at improving patient attitudes, rather than simply providing knowledge, could yield substantial improvements in self-management behaviors. From a clinical perspective, this implies that even when knowledge is adequate, without a corresponding positive attitude, practice behaviors may remain suboptimal. Attitude reflects patients’ emotional evaluation and behavioral inclination toward the disease and its treatment, including confidence in treatment, acceptance of the illness, and willingness to comply. A positive treatment attitude may enhance patients’ intrinsic motivation, making them more proactive in maintaining health behaviors. Thus, health education programs for osteomyelitis patients should allocate considerable resources to attitude change strategies, such as motivational interviewing and peer support, rather than focusing exclusively on information delivery. Third, attitude showed the strongest theoretical association with practice among all paths. This finding aligns with KAP studies from other regions and disease contexts. In South Africa, Masetla et al. reported that despite high self-reported adherence among patients with chronic bone and joint infections, most antibiotic prescriptions did not follow national guidelines, indicating a knowledge-practice gap similar to our observations (6). In Rwanda, Ndayisenga et al. found that 85% of patients delayed seeking care due to misconceptions and financial barriers, highlighting the critical role of attitudes and socioeconomic factors (7). Beyond Africa, studies in India on diabetic foot and in China on hypertensive nephropathy have similarly demonstrated that knowledge alone is insufficient to drive behavior change; attitude consistently emerges as a key mediator (32, 33). This provides a practical basis for integrating cognitive intervention and emotional support. Clinical interventions should not remain solely at the level of knowledge impartation. They also need to focus on patients’ psychological states and belief systems to improve negative emotions and compliance behaviors. These cross-cultural parallels suggest that the KAP framework and the mediating role of attitude are robust across different healthcare systems and diseases. However, our study is among the first to quantify this mediation in osteomyelitis using SEM, providing a more precise estimate of the attitude-mediated pathway. Future global collaborations could help determine whether these associations are universal or influenced by specific healthcare policies and cultural factors.

Based on the research findings, we propose the following clinical practice recommendations: establish a stratified health education system; develop tiered educational materials for patients with different education levels and ages — use illustrated manuals for those with low education levels and employ repeated reinforcement and family-involved education models for older patients; strengthen knowledge transfer during the initial diagnosis and treatment; ensure that first-time inpatients or outpatients receive systematic disease knowledge education, including core content such as osteomyelitis etiology, principles of antibiotic use, and the importance of follow-up; integrate psychological care with health education; use motivational interviewing and cognitive-behavioral interventions to help patients establish positive treatment attitudes; establish a continuity of care mechanism; provide ongoing knowledge support and behavioral reinforcement through telephone follow-up, mobile health apps, and similar means. Future interventions should integrate cognitive education, motivational enhancement, and continuous follow-up support to construct a bio-psycho-social integrated management model. In addition to patient-related factors, healthcare professionals’ awareness may also contribute to patients’ knowledge and practice levels. A recent study by Torun et al. (34). reported that insufficient physician awareness of chronic nonbacterial osteomyelitis was associated with diagnostic delays and inappropriate management strategies. Inadequate clinician familiarity with osteomyelitis-related conditions may reduce opportunities for effective patient education and communication, thereby indirectly affecting patients’ disease understanding and self-management behaviors. Future interventions should therefore target both patient education and healthcare professional training to improve overall disease management.

This study has several limitations. First, all participants were recruited from a single tertiary referral hospital. Patients attending tertiary healthcare institutions may have different disease characteristics, treatment experiences, and healthcare accessibility compared with patients in primary or secondary care settings. Therefore, the generalizability of our findings to broader osteomyelitis populations should be interpreted with caution. Future multicenter studies involving different healthcare settings are warranted. Second, the data were derived from patient self-reports, which may be subject to recall bias and social desirability bias; subsequent studies could incorporate objective indicators such as pharmacy records, electronic monitoring devices, or follow-up rates for verification. Third, important disease-specific characteristics, including infection severity, anatomical location, microbiological findings, antimicrobial treatment regimens, and surgical management strategies, were not available for analysis. These factors may influence patients’ knowledge acquisition, treatment experiences, and health behaviors. Future studies should incorporate detailed clinical variables to improve the clinical interpretability of KAP findings. Fourth, Although the questionnaire demonstrated excellent internal consistency, the very high Cronbach’s alpha coefficient may indicate partial overlap among some items. Future studies may further refine the instrument through item reduction and psychometric optimization. Fifth, although SEM provides a useful framework for examining theoretical relationships among KAP constructs, the cross-sectional design precludes causal inference. Therefore, the mediation findings should be interpreted as statistical associations that are consistent with, but do not prove, the hypothesized KAP framework.

## 5 Conclusion

This study found that knowledge, attitude, and practice levels among patients with osteomyelitis are generally low. Achievement rates for all three dimensions were below 40%. Age, education level, income level, and number of hospitalizations were the main factors associated with KAP. SEM results showed that knowledge had a direct theoretical association with practice and an indirect association through attitude. Attitude served as a key mediator in the knowledge-practice relationship. We recommend that clinical nursing work integrate health education and psychological support, implement personalized interventions targeting patients with low education, low income, and advanced age, and promote the formation and maintenance of health behaviors by improving attitudes. These efforts may enhance treatment adherence and long-term prognosis for patients with osteomyelitis.

## Data Availability

The original contributions presented in the study are included in the article/Supplementary material, further inquiries can be directed to the corresponding author/s.
