# Supplementary material for: Knowledge, attitude, and practice of patients with osteomyelitis: a cross-sectional study using structural equation modeling
Source: Front Public Health. 2026 Jul 10;14:1838790. doi: 10.3389/fpubh.2026.1838790 (PMC13396251; doi:10.3389/fpubh.2026.1838790)
Supplement: Supplementary file 1 [file Data_Sheet_1.docx]

| Questionnaire number: | |
| --- | --- |
| **Knowledge, Attitude and Practice of Patients with Osteomyelitis**  We are researchers from the Department of Orthopedics, Affiliated Hospital of Zunyi Medical Univeristy. We sincerely invite you to participate in our research project. This study aims to understand the knowledge, attitude, and practice toward osteomyelitis among patients diagnosed with this condition, to serve as the basis for developing scientific intervention strategies, which may help many others in the future to improve their health outcomes. Your participation in this study is voluntary, and the research has been approved by the Ethics Review Committee. If you agree to participate, please read the following instructions:   1. Please complete the questionnaire. There are no right or wrong answers; you only need to provide responses based on your actual experiences and understanding. If you have any questions during the process, feel free to reach out to us, and please submit the completed questionnaire in a timely manner. 2. This study is a simple questionnaire survey and will not cause any harm to your physical or psychological well-being. However, it may involve some personal information such as your gender, age, and disease-related details. Please rest assured that we will strictly maintain confidentiality and will not disclose your information. 3. As a participant, you can always stay informed about the information and progress related to this study. If you decide to withdraw from the study, please let us know, and your data will not be included in the research results.   Finally, we sincerely thank you for taking the time to support our scientific research amid your busy schedule.  □I have been informed and agree to the use of the collected data for scientific research.  Informed Consent Signature：  Date of participation： Year Month Day | |
| **Part I Basic Information**   \| **1.Your gender:** \| a. Male  b. Female \| \| --- \| --- \| \| **2.Your age(**years**):** \| a. ＜30  b. 30-50  c. 51-70  d. >70 \| \| **3. Your residence** \| a.Rural  b.Urban \| \| **4. Your** monthly income (RMB) \| a. ＜2000  b. 2000-5000  c. 5000-10000  d. 10000-20000  e. ＞20000 \| \| **5. Your** education level \| a. Junior high or below  b. High school/Vocational  c. Bachelor's or above \| \| **6.** Smoking? \| a. Yes  b. No \| \| **7.** History of diabetes? \| a. Yes  b. No \| \| 8. History of underlying disease/immunosuppression \| a. Yes  b. No \| \| 9. Surgery-related osteomyelitis \| a. Yes  b. No \| \| 10. Number of osteomyelitis-related hospitalizations \| a. 0  b. 1  c. 2  d. >3 \| | |

**Part II Knowledge of Patients with Osteomyelitis**

| Item | Very familiar | Familiar | Somewhat familiar | Not very familiar | very unfamiliar |
| --- | --- | --- | --- | --- | --- |
| 1. My understanding of the statement: "Osteomyelitis is a bacterial infectious disease of the bone." |  |  |  |  |  |
| 2. My understanding of the knowledge: "Advanced age (e.g., over 70 years) is a risk factor for developing osteomyelitis." |  |  |  |  |  |
| 3. My understanding of the knowledge: "Patients with diabetes are more susceptible to secondary osteomyelitis after minor foot or skin wounds." |  |  |  |  |  |
| 4. My understanding of the risk: "Postoperative surgical site infections in orthopedics can potentially extend deep into the bone, leading to osteomyelitis." |  |  |  |  |  |
| 5. My understanding of the knowledge: "Prolonged hospital stays increase the risk of nosocomial infections, including osteomyelitis." |  |  |  |  |  |
| 6. My understanding of the knowledge: "Individuals with underlying diseases like heart or kidney disease have lower resistance and are more prone to infections." |  |  |  |  |  |
| 7. My understanding of the knowledge: "An elevated C-reactive protein (CRP) level in blood tests often indicates infection or inflammation in the body." |  |  |  |  |  |
| 8. My understanding of the knowledge: "An elevated erythrocyte sedimentation rate (ESR) in blood tests is also commonly associated with infection or inflammation." |  |  |  |  |  |
| 9. My understanding of the knowledge: "The treatment course for osteomyelitis is typically very long and requires a full course of antibiotics." |  |  |  |  |  |
| 10. My understanding of the knowledge: "Treatment for osteomyelitis may require multiple surgeries for debridement and drainage." |  |  |  |  |  |
| 7*8+6=? | | | | | |

**Part III Attitude of Patients with Osteomyelitis**

| Item | Strongly agree | Agree | Uncertain | Disagree | Strongly disagree |
| --- | --- | --- | --- | --- | --- |
| 1. I believe osteomyelitis is a serious disease that must be given high priority. |  |  |  |  |  |
| 2. I am concerned that osteomyelitis is difficult to cure completely and may recur in the future. |  |  |  |  |  |
| 3. I believe that strictly controlling blood glucose is a key part of treating osteomyelitis. |  |  |  |  |  |
| 4. I believe that even after a surgical wound appears healed on the surface, hidden risks may still exist deep inside that require vigilance. |  |  |  |  |  |
| 5. I believe that accepting long-term hospitalization is necessary and worthwhile to cure osteomyelitis. |  |  |  |  |  |
| 6. I believe that actively managing underlying conditions like hypertension has a positive effect on the recovery from osteomyelitis. |  |  |  |  |  |
| 7. I believe that regular blood tests to monitor inflammatory markers (CRP, ESR) are very important for doctors to assess the condition. |  |  |  |  |  |
| 8. I trust that the treatment plan developed by my primary physician is scientific and effective. |  |  |  |  |  |
| 9. I am confident in my ability to overcome difficulties through perseverance and adhere to the entire treatment process. |  |  |  |  |  |
| 10. I believe that the patient's own attention to the disease and active cooperation are closely related to the treatment outcome. |  |  |  |  |  |
| 12*5+6= ? | | | | | |

**Part IV Practice on postoperative upper limb function exercise**

| Item | Always | Often | Sometimes | Rarely | Never |
| --- | --- | --- | --- | --- | --- |
| 1. I am able to strictly follow my doctor's advice, taking all medications on time and in the correct dosage. |  |  |  |  |  |
| 2. I return to the hospital for follow-up appointments punctually as scheduled by my doctor, never missing an appointment without reason. |  |  |  |  |  |
| 3. I regularly monitor my blood glucose and keep records. |  |  |  |  |  |
| 4. In daily life, I pay special attention to protecting the affected limb to prevent bumps or injuries. |  |  |  |  |  |
| 5. When I notice signs of infection in other parts of my body (e.g., toothache, boils), I immediately inform my doctor. |  |  |  |  |  |
| 6. I proactively observe the wound or affected area daily (e.g., for redness, swelling, pain, discharge). |  |  |  |  |  |
| 7. I consciously strengthen my nutrition through diet (e.g., increasing protein intake) to promote recovery. |  |  |  |  |  |
| 8. I will perform functional exercises as guided by my doctor to maintain joint and muscle function. |  |  |  |  |  |
| 9. I have started or am preparing to quit smoking, or have already reduced the amount I smoke. |  |  |  |  |  |
| 10. When I have questions about my treatment or rehabilitation, I actively seek answers and help from healthcare staff. |  |  |  |  |  |
| 8*8-5=? | | | | | |
